# Safety Profile of Sclerosing Agents in the Management of Low‐Flow Vascular Malformations of the Head and Neck—A Systematic Review

**DOI:** 10.1002/hed.70338

**Published:** 2026-06-04

**Authors:** Riccardo Nocini, Carlotta Muneretto, Athena E. Arsie, Valerio Arietti, Benedetta Lorenzon, Alessandra Cinotti, Giacomo Colletti

**Affiliations:** ^1^ Head and Neck Department, Unit of Otorhinolaryngology University of Verona Verona Italy; ^2^ Cranio‐Maxillo‐Facial Surgery, SMECHIMAI Department University of Modena and Reggio Emilia Modena Italy

**Keywords:** head and neck, sclerosing agents, sclerotherapy complications, vascular malformations, venous and lymphatic malformations

## Abstract

**Background:**

Low‐flow vascular malformations (LFVMs) of the head and neck, including venous and lymphatic malformations, represent a heterogeneous group of congenital anomalies frequently requiring intervention due to functional and esthetic impairment. Percutaneous sclerotherapy has emerged as a first‐line treatment; however, no consensus exists regarding the optimal sclerosant, and all agents are associated with potential complications. This systematic review aims to evaluate and compare the safety profiles of commonly used sclerosing agents in the management of LFVMs of the head and neck.

**Methods:**

A systematic review was conducted according to PRISMA guidelines. PubMed, Scopus, and Google Scholar were searched for studies published between 2005 and 2025 reporting complications of sclerotherapy for LFVMs of the head and neck. Inclusion criteria comprised clinical studies with patients treated with intralesional sclerotherapy as a single modality and reporting treatment‐related complications. Data extraction focused on type of malformation, sclerosant used, number of sessions, and complication rates per session. Complications were categorized as local or systemic and further stratified into minor and major events.

**Results:**

A total of 64 studies encompassing 2508 patients and 5193 sclerotherapy sessions were included. The overall complication rate was 11.2% per session, with the majority being minor local events (8.8%). Bleomycin was the most frequently used agent (1516 sessions; 13.5% complication rate), predominantly associated with mild local and systemic inflammatory reactions. Ethanol (1072 sessions; 9%) demonstrated higher rates of major local complications (1.7%), including tissue necrosis and permanent nerve injury, as well as systemic effects such as hemoglobinuria and rare cardiopulmonary events. Sodium tetradecyl sulfate (STS) (1061 sessions; 9.5%) showed a moderate complication profile but carried risks of necrosis and airway edema. Polidocanol (860 sessions; 4.2%) and doxycycline (158 sessions; 3.8%) exhibited the most favorable safety profiles, with no major complications reported. Ethanolamine oleate had the highest complication rate (20.2%), including significant local adverse events.

**Conclusions:**

Sclerotherapy for LFVMs of the head and neck is generally safe, with most complications being minor and self‐limiting. However, the safety profile varies significantly among sclerosants. Given the absence of standardized guidelines and comparative efficacy data, safety considerations should play a central role in sclerosant selection.

## 1 Introduction

Vascular anomalies represent one of the most frequent congenital and neonatal dysmorphogenetic conditions, encompassing a broad spectrum of disorders. They may arise in any anatomical region, although approximately 60% are localized within the head and neck. Historically, these entities were often grouped under the generic term haemangioma. In 1982, Mulliken and Glowacki proposed a classification that distinguished vascular tumors from congenital vascular malformations, relying on clinical features, histopathology, and histochemical characteristics [1]. This system was subsequently endorsed by the International Society for the Study of Vascular Anomalies (ISSVA) during the 1996 workshop held in Rome, where the distinction between vascular tumors (endothelial proliferation) and vascular malformations (developmental defects in angio‐ or lymphangiogenesis) was formally recognized [2]. The revised classification proposed by Waner and Suen is considered more precise and clinically applicable. In this system, vascular malformations are categorized into venular malformations, venous malformations, arteriovenous malformations, microcystic and macrocystic lymphatic malformations, as well as mixed forms, depending on their structural components [3]. The 2025 ISSVA classification retains the fundamental division of vascular anomalies into vascular tumors and vascular malformations, the latter being further categorized based on hemodynamic behavior into slow‐flow, fast‐flow, and anomalies involving major named vessels. A newly introduced category, “potentially unique vascular anomalies (PUVA),” encompasses lesions that are not yet fully defined. This updated classification also emphasizes the integration of clinical, imaging, and molecular‐genetic features, supporting a more biologically oriented and adaptable framework [4]. This classification has been proven valuable in guiding therapeutic decision‐making.

Slow flow malformations, although theoretically involving capillary malformations too, encompass venous and lymphatic malformations.

Venous malformations (VMs) probably represent the most common type of vascular malformation. Approximately 40%–60% of VMs occur in the head and neck region. Venous malformations result from mosaic somatic mutations (or very rarely germinal) involving TIE2/TEK or rarely PIK3CA. These alterations disrupt the balance between vascular differentiation and angiogenesis, leading to abnormal endothelial signaling and defective development of the tunica media. The absence or disorganization of smooth muscle support makes the vessels prone to progressive dilatation, often extending into surrounding tissues. This structural weakness also accounts for the variability in lesion size depending on body position. Within the ectatic venous channels, blood flow is markedly reduced, predisposing to stasis, thrombin activation, and intralesional thrombosis, a process described as localized intravascular coagulation (LIC). Clinical presentation is variable and may include cosmetic disfigurement, pain, swelling, and functional impairment. In a small minority of cases, the condition is mild and can be managed conservatively without the need for active intervention. Most often, symptoms are severe enough to require multiple treatment sessions, often necessitating progressively invasive approaches [5].

Congenital lymphatic malformations (LMs) have an estimated annual incidence of approximately 1 in 5000 live births, with around 75% of cases occurring in the head and neck region [6]. These lesions are caused by a somatic mosaic mutation in the PIK3CA gene (but complex lymphatic malformations can harbor mutations in the RAS‐MAP pathway). This causes the inability of endothelial cells to build a proper lymphatic system and instead of channels is made up by chambers, called cysts. Histologically, LMs are classified as microcystic (lymphatic spaces < 1 cm), macrocystic (lymphatic spaces > 1 cm), or mixed types [7]. By the age of 2 years, up to 90% of lymphatic malformations become clinically apparent [8]. Although the lesions are nonproliferative, they may enlarge over time due to the gradual accumulation of lymphatic fluid. Delayed or initially hidden presentation often becomes evident following a sudden increase in size, typically triggered by spontaneous hemorrhage or infection of the cystic contents, frequently associated with even minor infections. Spontaneous regression occurs only anecdotally, and in the absence of treatment, complications such as infection or hemorrhage may arise, potentially exerting pressure on adjacent head and neck structures, including the airway [9, 10].

A wide range of therapeutic approaches for slow flow vascular anomalies has been reported in the literature, including laser ablation, sclerotherapy, electrochemical therapy, copper needle application, surgical resection, and multimodal strategies. Recently, targeted medical treatment has also become available and is the preferred treatment in some cases. The choice of treatment is influenced by factors such as lesion type, anatomical location, size, patient condition, and the available expertise. While surgical excision has historically been the preferred modality for vascular malformations of the head and neck, today a multimodal approach is usually adopted. When lesions infiltrate or encase critical structures, such as the facial nerve, complete excision is challenging, and less invasive therapeutic strategies are preferable to avoid iatrogenic injury. Percutaneous sclerotherapy is regarded as the technique of choice in many instances owing to its relatively reduced invasiveness. This technique has demonstrated safety and efficacy in the management of small‐ to medium‐sized malformations and has also been employed as a preoperative adjunct for larger lesions, helping to reduce intraoperative blood loss and to better define surgical margins.

A variety of sclerosing agents is described in the literature including alcohol, detergents, hyperosmotic solutions, and antineoplastic agents.

Bleomycin (a macrolide antibiotic) was initially introduced as an antineoplastic agent due to its ability to inhibit DNA synthesis. Subsequent evidence demonstrated that the drug also exerts a sclerosing effect on endothelial cells, mediated by a nonspecific inflammatory response [11]. Bleomycin has been successfully used for the primary therapy of both microcystic and macrocystic lymphatic malformations and venous malformations with very good effect [12, 13].

Pingyangmycin, also known as bleomycin A5, is a compound developed in China and derived from various components of bleomycin synthesized by Streptomyces pingyangensis. While its overall chemical structure closely resembles that of bleomycin, it differs in the composition of the terminal amine group [14]. The drug targets rapidly proliferating cells by interfering with the G2 and S phases of the cell cycle and inducing single‐strand DNA breaks. It also impairs DNA repair processes through inhibition of DNA ligase. When administered intralesional, Pingyangmycin comes into direct contact with the endothelial lining, leading to endothelial cell damage and resulting in sclerosis and narrowing of the vessel lumen [15]. Histological observations following injection demonstrate endothelial cell swelling and vacuolization, disruption of the tunica intima, thickening of the vascular walls, luminal narrowing, and eventual occlusion [16].

Ethanol sclerotherapy has been shown to be effective in the management of venous malformations (VMs). It exerts its effect by directly damaging the intima through a combination of direct cytotoxic action on the vascular wall and aggregation of injured erythrocytes with denatured proteins, resulting in permanent vessel lumen occlusion [17, 18]. Once injected intravascularly, ethanol denatures plasma proteins, induces dehydration of endothelial cells, precipitates their cytoplasmic contents, and strips the vascular wall of its endothelial lining, sometimes producing fractures that extend to the internal elastic lamina [19, 20]. Vascular spasm together with perivascular necrosis promotes the formation of acute thrombi [21].

Doxycycline, a member of the tetracycline antibiotic class, was first reported for the management of lymphatic malformations by Molitch et al. [22]. Although its precise sclerosing mechanism has not been fully elucidated, its activity has been linked to the inhibition of matrix metalloproteinases and cellular proliferation, as well as to the suppression of vascular endothelial growth factor involved in angiogenesis and lymphangiogenesis. These effects promote collagen and fibrin deposition, ultimately resulting in the development of dense adhesions and fibrosis [23]. A direct irritating effect also translates in endothelial cells' damage.

Sodium tetradecyl sulfate (STS), commercially known in the US as sotradecol, is an anionic surfactant that damages the endothelial cell membrane by altering the lipid bilayer structure and denaturing membrane‐associated proteins, including clotting factors. These effects trigger fibrosis and result in vascular occlusion [24]. Sclerotherapy using STS can be conducted safely in an outpatient setting in selected cases of limited VMs. The agent may be administered either in its liquid form or as a foam prepared with air using the Tessari technique [25].

OK‐432 (OKama Strain‐432, Picibanil) is a lyophilized preparation of an inactive‐non infectious strain of *Streptococcus pyogenes* group A, cultured in the presence of benzylpenicillin [26]. The proposed mechanism of action of this pro‐inflammatory immunostimulant involves the induction of various cytokines. The resulting inflammatory response remains localized but is sufficient to induce endothelial damage [27].

Polidocanol/Lauromacrogol (Aethoxysclerol) classified as a nonionic detergent produces its sclerosing effect by damaging the endothelial lining through membrane lysis and consequent endothelial destruction [24].

Ethanolamine oleate is an emulsion of fatty acids widely employed in the past as a sclerosing agent in the management of vascular malformations. Its mechanism of action is based on the induction of thrombosis, which subsequently causes endothelial injury and leads to progressive obliteration of the vascular lumen [28]. Oleic acid has been shown to promote coagulation by stimulating tissue factor release and activating Hageman factor; however, a significant pro‐coagulant effect is generally not observed, likely because the ethanolamine fraction interferes with fibrin clot formation through calcium chelation [29].

Currently, there is no consensus as to the best sclerosant to use in a given clinical scenario, and all are associated with complications. Because venous malformations (VMs) are relatively rare, occurring in roughly 1–2 per 10 000 live births with a prevalence near 1% [30, 31], conducting a randomized trial to compare different sclerosing agents is considered unlikely. Moreover, a standardized outcome measure for assessing the response of vascular malformations to sclerotherapy has not yet been established, and the currently available literature shows considerable heterogeneity in reporting treatment results. This lack of uniformity significantly limits an accurate evaluation of therapeutic efficacy and makes it difficult to compare the effectiveness of different sclerosing agents.

This systematic review is focused on assessing the complications related to each specific sclerosing agent. Establishing the safety profiles of these drugs, given the current lack of standardized guidelines for the management of vascular malformations, and the absence of definitive evidence supporting the superior efficacy of one agent over another, may serve as a critical tool in guiding the selection of the safest and, consequently, the most appropriate sclerosant.

## 2 Methods

A systematic literature search was performed in PubMed, Scopus, and Google Scholar to identify relevant studies reporting complications associated with sclerotherapy for low‐flow vascular malformations. The review was conducted in accordance with the Preferred Reporting Items for Systematic Reviews and Meta‐Analyses (PRISMA) guidelines. The following search string was applied: ((venous malformation OR venous anomaly OR lymphatic malformation OR lymphatic anomaly OR lymphatic abnormalities) AND (head and neck OR craniofacial OR facial OR oral OR oropharyngeal) AND (complications OR adverse effects OR sequelae OR bleeding OR hemorrhage OR infection OR airway obstruction OR pain OR ulceration)) AND (2005:2025[dp]).

All titles and abstracts were assessed; the full texts of relevant studies were screened for final selection. All studies identified by the initial literature search were reviewed independently by three authors.

### 2.1 Eligibility Criteria

**Table:** 

Inclusion	Exclusion
Studies published between 2005 and 2025	No full text or abstract available
RCTs and observational studies (case series or cohort studies)	Nonhuman studies (animal or in vitro)
Low‐flow vascular malformations (venous and lymphatic) of the head and neck	Case series including lesions located in other anatomical region than the head and neck without subsite‐specific data on treatment and complications
> 5 patients treated with intralesional sclerotherapy	Case reports with < 5 patients or review articles
Sclerosant therapy as sole treatment modality*	Studies on vascular tumors (angioma/hemangioma) or high‐flow vascular malformations (arteriovenous malformations)
Type of sclerosing agent used and number of treatment sessions performed per patient reported	Studies without clear reporting of treatment modality or complications
Treatment‐related complications reported	Multimodal treatments where complications from sclerotherapy could not be isolated

* In studies describing multiple treatment modalities (including surgery, laser therapy, or others), only data pertaining to patients treated with sclerotherapy alone were included.

Flow diagram of systematic literature search and study selection, according to PRISMA is reported.

Previous treatments were not considered, as the objective was to evaluate complications related to the individual procedure (rate of complication per sclerotherapy session). All complications were gathered and grouped into the following categories:

**Table:** 

Local complications
*Minor*
Injection‐site complications (minor edema, pain, perilesional erythema)
Minor local complications (superficial ulcerations, aphthae, minor local infection)
Cosmetic alteration at the treated site (scarring)
Skin discoloration (hypo‐ or hyperpigmentation)
Transient sensory deficit (hypoesthesia/paresthesia)
Transient motor nerve deficit (< 1 month)
Hemorrhagic complications (hematoma, self‐limiting minor bleeding)
*Major*
Major local complications (necrosis with tissue lost)
Long lasting motor nerve deficit (> 1 month)
Airways complications (oedema requiring tracheostomy or prolonged intubation)

Systemic complications
*Minor*
Mild allergic reactions (rash/pruritus)
Systemic inflammatory reaction (fever)
Nausea/vomiting
Hemoglobinuria (without renal faillure)
*Major*
Severe allergic reactions (anaphylaxis)
Cardiocirculatory complications

Data were extracted by a single independent author and collected in Excel.

To have a complete and meticulous picture of severe complications, single case reports of catastrophic events are also reported and discussed.

## 3 Results

Among 64 included articles, 2508 patients' data were evaluated. Of the 64 articles reviewed, 17 focused exclusively on lymphatic malformations (362 patients), 42 on venous malformations (1768 patients), and 5 included mixed cohorts of venous and lymphatic malformations (378 patients: 304 venous and 74 lymphatic). Of the 64 articles reviewed, 62 exclusively reported on lesions confined to the head and neck region. In the remaining two studies, lesions were also present in other anatomical sites; however, only patients with disease localized to the head and neck were included in the data analysis. In 51 articles, sclerotherapy was the sole treatment modality reported. In 13 articles, patients underwent multimodal treatments (including surgery, radiofrequency, and other interventions); however, for the purpose of data analysis, only those patients treated with sclerotherapy alone were considered.

**Figure:**
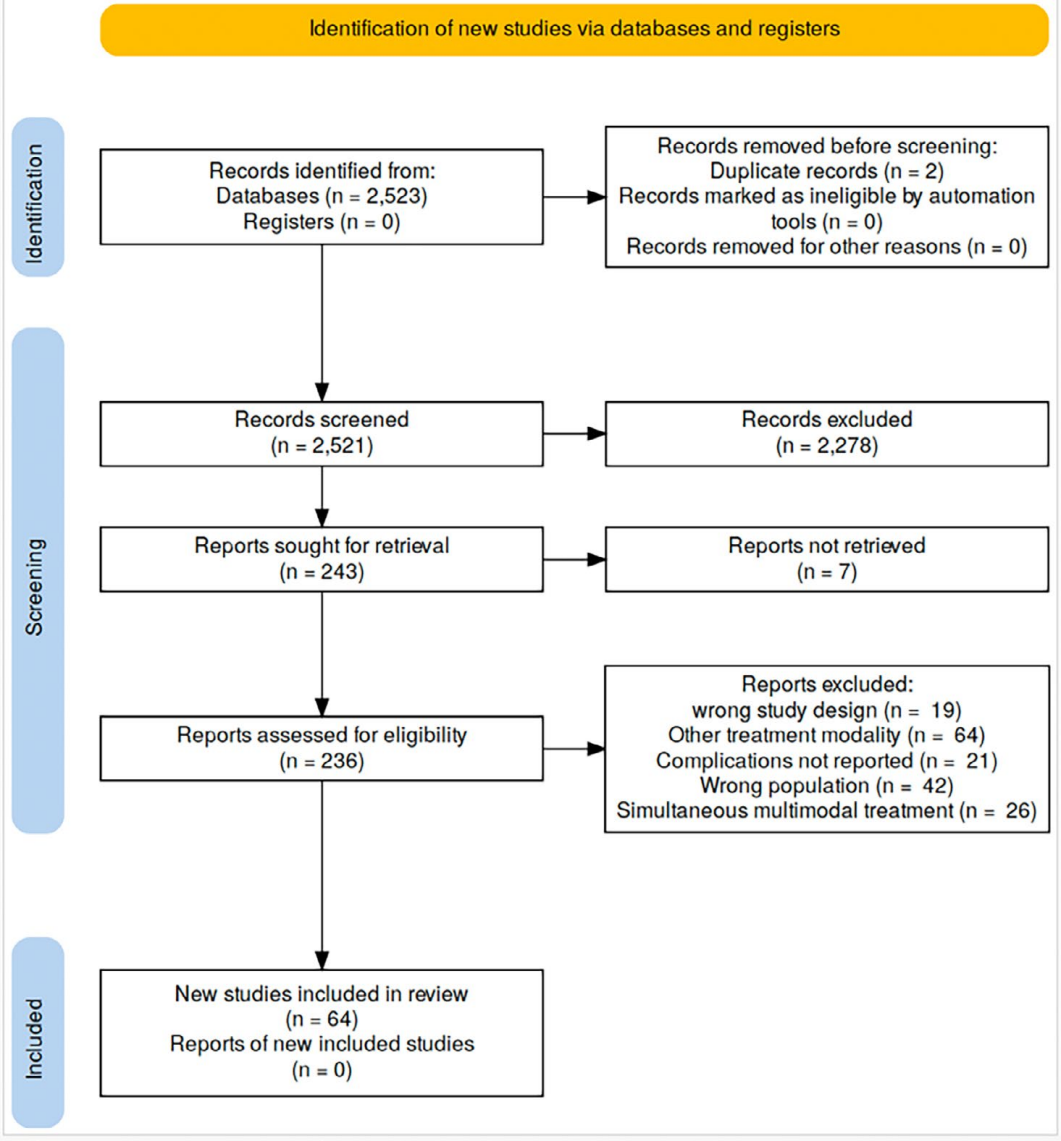
[Color figure can be viewed at wileyonlinelibrary.com]

Table 1 summarizes the key characteristics of the studies included in this review. For each article, the table outlines the sample size, the specific type of vascular malformation evaluated, the sclerosant agent, and the number of sclerotherapy sessions performed. It also reports the incidence of complications documented across the included cohorts.

**TABLE 1:** Included articles.

Author	Year	Country	No of patients	Type of malformation	No of scleroterapy session	No of complication	Complication rate	Sclerosing agents
Jia Wei Zheng [14]	2009	China	297	VM, LM	297	5	1.71%	Bleo
D. Wook Kim [27]	2014	Korea	26	LM	37	0	0.00%	Pici
M. D. Alexander [28]	2015	USA	26	VM	52	0	0.00%	Etan
Rui Hou [32]	2010	China	75	VM	123	51	57.72%	Bleo
W. L. Chen [33]	2007	China	16	VM	64	19	29.69%	Bleo + pici
P. A. Valletti [34]	2019	Germany	9	LM	9	0	0.00%	Pici
Lixin Su [35]	2010	China	60	VM	156	11	7.05%	Alc
Da‐Peng Xu [36]	2014	China	32	LM	51	1	1.96%	Bleo
Yaowu Yang [37]	2011	China	65	LM	195	8	4.10%	Bleo
Ul Haq [38]	2015	USA	17	VM	17	8	42.11%	Bleo; bleo + STS; bleo + alc
T. Shigematsu [39]	2018	USA	18	VM	56	18	32.14%	Bleo
N. N. Mathur [40]	2005	India	10	LM	36	7	19.44%	Bleo
Yang Jiang [41]	2023	China	26	LM	52	5	9.62%	Bleo
Yu Liu [42]	2009	China	23	VM	58	56	100.00%	Bleo + alc
Bajpai [43]	2012	India	16	VM	49	9	18.37%	Bleo; STS
P. Shivhare [44]	2022	India	20	VM, LM	32	0	0.00%	STS
G. Colletti [45]	2017	Italy	69	VM	148	60	41.89%	STS
D. Grieb [46]	2018	Germany	20	VM	56	1	1.79%	Poli
Xing Wang [47]	2016	China	22	VM	56	11	19.64%	Alc
J. Meng [48]	2014	China	43	VM	43	6	13.95%	Bleo + alc
Jiapeng Li [49]	2010	China	20	VM	72	9	12.50%	Bleo; alc
Deming Wang [50]	2016	China	21	VM	50	27	54.00%	Alc
Hye Jin Baek [51]	2011	Korea	22	VM	37	2	5.41%	Alc
Depika Nehra [52]	2008	USA	11	LM	23	0	0.00%	Doxy
Olivia Maleux [53]	2022	Belgium	27	LM	108	5	4.81%	Doxy
H. A. Helal [54]	2019	Egypt	31	VM	75	1	1.33%	Bleo
M. C. Ribeiro [55]	2018	Brasil	34	VM	96	8	8.33%	Etan
Hao Zhang [56]	2019	China	28	VM	84	21	25.00%	Bleo
Z. Sun [57]	2024	China	223	VM	380	3	0.80%	Poli
K. Katayama [58]	2024	Japan	16	VM	22	0	0.00%	Poli
A. ‐W. Chen [59]	2015	China	11	VM	43	1	2.33%	Poli
In Ho Lee [60]	2009	Korea	87	VM	305	4	1.31%	Alc
O. P. Caldas [61]	2014	Brasil	51	VM	357	9	2.63%	Alc
I. Maeda [62]	2024	Japan	14	VM	48	1	2.08%	Poli
S. Kumar [63]	2020	India	17	VM	35	2	5.71%	Poli
D. F. Vollherbst [64]	2020	Germany	27	VM	51	18	41.18%	Alc; poli
H. M. Mai [65]	2011	China	18	LM	57	44	105.56%	Bleo
Keyao Li [66]	2023	China	52	VM	52	7	13.46%	Poli
Hanwen Chu [67]	2019	China	22	VM	22	18	81.82%	Bleo
L. A. Benoiton [68]	2017	N.Z.	6	VM	12	2	16.67%	Alc
K. W. Rosbe [69]	2010	USA	10	VM	10	0	0.00%	STS
Kento Suzuki [70]	2014	Japan	22	LM	33	0	0.00%	Poli
S. J. Boardman [71]	2010	UK	21	LM	41	8	19.51%	Pici; alc
R. Dasguptaa [72]	2008	USA	7	LM	10	1	10.00%	Alc; doxy
Hui Chen [73]	2008	China	15	VM	18	2	11.11%	Alc
S. Krings [74]	2010	Canada	31	VM	111	4	3.60%	Bleo
W. ‐L. Chen [75]	2011	China	15	LM	15	1	6.67%	Bleo + pici
W. L. Chen [76]	2009	China	18	VM	18	2	11.11%	Bleo + pici
Kenneth Kok [77]	2012	UK	21	VM, LM	23	6	26.09%	STS
Ierardi [78]	2019	Italy	6	VM, LM	6	3	50.00%	Alc
J. Spence [79]	2011	Canada	34	LM	88	9	10.34%	Bleo; alc
A. Sindel [80]	2018	Turkey	34	VM, LM	34	4	11.76%	Bleo
S. Y. Kim [81]	2015	Korea	9	LM	9	4	44.44%	Pici
An‐Wei Chen [82]	2018	China	70	VM	150	1	0.67%	Poli
Yaowu Yang [83]	2008	China	7	VM	7	2	28.57%	Bleo
Zeevi [84]	2020	Israel	25	VM	40	24	72.50%	Etan
Y. Awang [85]	2009	China	23	VM	23	4	17.39%	Alc
D. A. Shaye [86]	2020	Rwanda	10	LM	21	1	4.76%	Etan
Paul Stimpson [87]	2011	UK	12	VM	36	1	2.78%	STS
Alakailly [88]	2015	USA	13	VM	13	3	23.08%	STS
Jan Shah [89]	2022	India	30	VM	105	15	14.29%	Bleo
E. Karimi [90]	2018	Iran	345	VM	759	18	3.05%	STS
Dror Gilonya [91]	2012	Israel	20	LM	25	2	8.00%	Pici
Keqian Zhi [92]	2008	China	82	VM	82	8	9.76%	Bleo

Abbreviations: alc, ethanol/alcohol; bleo + alc, co‐administration of bleomycin and ethanol; bleo + pici, co‐administration of bleomycin and picibanil; bleo + STS, co‐administration of bleomycin and sodium tetradecyl sulfate; Bleo, bleomycin/pingyangmycin; doxi, doxycycline; etan, ethanolamine oleate; LM, lymphatic malformation; pici, picibanil/OK‐432; poli, polidocanol; STS, sodium tetradecyl sulfate; VM, venous malformation.

Across a total of 2508 patients 5193 sclerotherapy sessions were performed. The distribution of sclerosing agents in the analyzed cohort, complication type and complication rate per session for each sclerosing agent are reported in Table 2.

**TABLE 2:** Complications per sclerosing agent.

Sclerosant	No of session	No of complication	Complication rate	Minor local	Major local	Minor sistemic	Major sistemic
Total	5193	580	11.2%	455 – 8.8%	41 – 0.8%	78 – 1.5%	6% – 0.1%
Bleo	1516	204	13.5%	171 – 11.3%	2 – 0.1%	31 – 2%	0
Alc	1072	96	9%	41 – 3.8%	19 – 1.7%	33 – 3.1%	3% – 0.3%
Doxi	158	6	3.8%	6 – 3.4%	0	0	0
STS	1061	101	9.5%	89 – 8.3%	10 – 0.9%	0	2 – 0.2%
Poli	860	36	4.2%	30 – 3.5%	0	6 – 0.7%	0
Pici	116	10	8.6%	6 – 5.2%	0	4 – 3.5%	0
Etan	188	38	20.2%	29 – 15.4%	9 – 4.8%	0	0
Bleo + alc	123	67	54.5%	61 – 49.5%	1 – 0.8%	4 – 3.3%	1 – 0.8%
Bleo + STS	2	0	0%	0	0	0	0
Bleo + pici	97	22	34.4%	22 – 34.4%	0	0	0

Abbreviations: alc, ethanol/alcohol; bleo + alc, co‐administration of bleomycin and ethanol; bleo + pici, co‐administration of bleomycin and picibanil; bleo + STS, co‐administration of bleomycin and sodium tetradecyl sulfate; Bleo, bleomycin/pingyangmycin; doxi, doxycycline; etan, ethanolamine oleate; pici, picibanil/OK‐432; poli, polidocanol; STS, sodium tetradecyl sulfate.

The breakdown of complications by individual sclerosing agent and the corresponding complication rates per sclerotherapy session are presented in Table 3. In six studies, two agents were co‐administered within the same procedure, precluding the attribution of specific complications to a single agent. Complications associated with the combined administration of both agents are also reported.

**TABLE 3:** Complications per sclerosing agents details.

	Bleo	Alc	Doxy	STS	Poli	Pici	Etan	Alc + bleo	Bleo + STS	Picin + bleo
Injection‐site complications	94 – 6%	8 – 0.8%	2 – 1.3%	21 – 2%	20 – 2.3%	6 – 5.2%	23 – 12.2%	58 – 47%	—	16 – 25%
Transient sensory deficit	—	7 – 0.7%	—	—	—	—	—	—	—	—
Motor nerve deficit	1 – 0.06%	15 – 1.4%	1 – 0.6%	—	—	—	—	—	—	1 – 1.6%
Minor local complications	67 – 4.4%	8 – 0.8%	1 – 0.6%	57 – 5.4%	4 – 0.5%	—	—	3 – 2.4%	—	—
Major local complications	—	15 – 1.4%	—	7 – 0.7%	—	—	9 – 4.8%	—	—	—
Airways complications	2 – 0.1%	2 – 0.2%	—	3 – 0.3%	—	—	—	1 – 0.8%	—	—
Cosmetic alteration at the treated site	2 – 0.1%	3 – 0.3%	—	1 – 0.1%	2 – 0.2%	—	—	—	—	1 – 1.6%
Skin discoloration	4 – 0.3%	2 – 0.2%	—	—	—	—	—	—	—	4 – 6.2%
Hemorrhagic complications	3 – 0.2%	—	2 – 1.3%	10 – 0.9%	4 – 0.5%	—	6 – 3.2%	—	—	—
Mild allergic reactions	2 – 0.1%	—	—	—	—	—	—	—	—	—
Severe allergic reactions	—	—	—	1 – 0.1%	—	—	—	—	—	—
Systemic inflammatory reaction	26 – 1.7%	5 – 0.5%	—	—	5 – 0.6%	4 – 3.5%	—	3 – 2.4%	—	—
Nausea/vomiting	3 – 0.2%	5 – 0.5%	—	—	1 – 0.1%	—	—	—	—	—
Hemoglobinuria	—	23 – 2.2%	—	—	—	—	—	1 – 0.8%	—	—
Cardiocirculatory complications	—	3 – 0.3%	—	1 – 0.1%	—	—	—	1 – 0.8%	—	—

Abbreviations: alc, ethanol/alcohol; atos, Aethoxysklerol/lauromacrogol; bleo + alc, co‐administration of bleomycin and ethanol; bleo + pici, co‐administration of bleomycin and picibanil; bleo + STS, co‐administration of bleomycin and sodium tetradecyl sulfate; Bleo, bleomycin/pingyangmycin; doxi, doxycycline; etan, ethanolamine oleate; pici, picibanil/OK‐432; poli, polidocanol; STS, sodium tetradecyl sulfate.

Reported cardiocirculatory complications included one case of ophthalmic vein thrombosis and two episodes of transient desaturation associated with ethanol use; one paradoxical embolism in the setting of a patent foramen ovale following STS administration (not considered directly attributable to the sclerotherapy procedure by the authors but nonetheless documented); and one episode of self‐limited chest pain occurring after co‐administration of ethanol and bleomycin.

Details regarding motor nerve deficits are reported in Table 4. The only two permanent paralyzes were observed following ethanol use, specifically one facial nerve palsy and one recurrent laryngeal nerve palsy. All other nerve palsies resolved spontaneously within 4 weeks.

**TABLE 4:** Motor nerve deficit.

Sclerosant	Permanent motor nerve deficit	Transient motor nerve deficit
Bleomycin/pingyangmycin	0	1
Ethanol/alcohol	2	13
Doxycycline	0	1
Bleo + pici	0	1

## 4 Discussion

Our findings indicate that bleomycin represents the most commonly employed sclerosing agent for the management of low‐flow vascular malformations of the head and neck region, followed by ethanol and sodium tetradecyl sulfate. Additional agents considered within the review included doxycycline, polidocanol, picibanil, and ethanolamine oleate. Ethanol, polidocanol, and ethanolamine oleate were utilized solely for the management of VMs, whereas doxycycline and OK‐432 were reserved for LMs. The other sclerosing agents were applied to both malformation types.

Ethanolamine oleate sclerotherapy is associated with a high complication rate, with serious local adverse events such as skin and tissue necrosis (4.8% per sclerotherapy session, as shown in Table 3). Given the presence of critical anatomical structures in the head and neck, skin ulceration or necrosis in this region can be particularly debilitating and for this reason we recommend caution.

STS has also been associated with an increased risk of tissue necrosis, as well as a higher likelihood of significant oedema potentially leading to airway obstruction when compared with other sclerosing agents 0.7% and 0.3% per sclerotherapy session, respectively, as reported in Table 3. Allevi F. et al. reported a case of a woman with a large cervicofacial venous malformation treated with fluoroscopy‐guided intralesional foam sclerotherapy using STS and air under general anesthesia. Upon awakening, she exhibited dysarthria and dysmetria. Brain CT revealed no haemorrhagic lesions, and chest CT excluded pulmonary embolism. A paradoxical embolism was suspected, and echocardiography confirmed a patent foramen ovale, with transcranial ultrasound showing mild right‐to‐left shunting. The neurological deficits resolved spontaneously within 48 h, and follow‐up MRI showed no ischemic or haemorrhagic lesions [93]. No cases of paradoxical embolism were reported among the studies included in our review; however, in light of potential similar events, caution is advised in patients with comparable cardiac conditions. Another potential risk, although not observed in the studies included in our review, is ischemic injury due to arterial occlusion. E. Castren et al. reported a case of a palatal venous malformation treated with STS, complicated by occlusion of the peripheral vascular bed of the internal maxillary artery in the palatine region, caused by accidental intra‐arterial injection of the sclerosant. This led to bone necrosis, necessitating partial surgical resection of the maxilla, resulting in a permanent bone defect and allodynia [94]. This potential risk is not specific to STS, and caution must be exercised regarding the correct injection site to prevent similar sequelae even with other sclerosing agents.

With ethanol use, cases of airway edema requiring tracheotomy or prolonged intubation and tissue necrosis have been documented in our review (0.2% and 1.4%, respectively, as shown in Table 3). Compared with other embolic agents, ethanol possesses several advantageous features for treating VMs: as a liquid embolic material, it can diffuse throughout the venous system; it is inexpensive; and its metabolic pathways and excretion in humans are well established. Because ethanol achieves complete destruction of endothelial cells, recanalization and neovascularization are rarely observed [95, 96, 97]. However, the therapeutic index of absolute alcohol is utterly narrow. Reported complications are primarily related to extravasation, which can lead to necrosis of surrounding soft tissues. Similarly, inadvertent nontarget injection results in tissue necrosis due to obliteration of capillary networks. When using the direct puncture technique, precise positioning of the needle within the malformation is critical prior to injection, as accidental extravasation from displacement can cause severe damage. For this reason, fluoroscopic guidance is recommended during the procedure [35]. However, the only severe complication with ethanol in our review was permanent motor nerve paralysis (as shown in Table 4). Consequently, we advise cautiouos use of ethanol for sclerotherapy in the cervicofacial area, especially for superficial lesions or those in close proximity to nerves. In our review haemoglobinuria has been observed solely in association with ethanol‐based therapy, including cases combined with bleomycin, yet it was not accompanied by any instances of renal failure and not considered as a severe event.

In our results, two episodes of transient desaturation associated with ethanol use were observed. Although in the series by In Ho Lee et al. [60] included in our review these events were mild and self‐limiting, the literature reports cases of severe cardiopulmonary events associated with ethanol treatment. Ethanol‐induced intravascular hemolysis may trigger pulmonary vasoconstriction via nitric oxide depletion resulting in acute pulmonary hypertensive crisis. Cordero‐Schmidt et al. described a case of acute pulmonary hypertensive crisis during the treatment of malformation involving the face and orbit [98]. Rimon et al. reported severe cardiopulmonary complications in a series of 21 patients with malformations predominantly of venous origin. In one patient, acute pulmonary hypertension with cardiovascular collapse occurred after a large ethanol dose (1 cc/kg), requiring resuscitation and 24 h in the ICU; the patient was discharged 3 days later and underwent four subsequent treatments without complications. Another patient developed a pulmonary embolism 2 days after sclerotherapy for a right calf malformation; a hypercoagulable state was identified, and full recovery ensued without sequelae [99]. Wong et al. described a case of cardiovascular collapse characterized by sudden tachypnea, oxygen desaturation, and bradycardia, with loss of peripheral pulses. Evidence of right ventricular and pulmonary artery hypertension was noted. The patient regained consciousness in the operating room after 45 min after resuscitative maneuvers. She was transferred intubated and on dopamine to the ICU, where she was weaned and extubated the same afternoon. Follow‐up echocardiography 10 days later demonstrated normal biventricular function without regional wall motion abnormalities; no long‐term sequelae were reported [60]. Chapot et al. described a fatal cardiovascular collapse following ethanol sclerotherapy for a venous malformation in the anterior thigh. The patient died after 4 h of resuscitation. Autopsy revealed no macroscopic lesions of the heart or lungs and no pulmonary embolism. Death was attributed to ethanol‐induced cardiac toxicity, likely triggered by a bolus effect following tourniquet release [100].

Since direct pulmonary arterial pressure monitoring is invasive and not routinely employed, early recognition of such complications depends on clinical vigilance, with intraoperative echocardiography providing valuable support. Procedural strategies should aim to minimize pulmonary hypertension by ensuring adequate anesthesia, hyperoxia, and mild hyperventilation, and by maintaining readiness for nitric oxide administration. The presence of an anesthesiology team ready to initiate resuscitative maneuvers is of crucial importance.

In our analysis, bleomycin and pingyangmycin were aggregated due to their close pharmacological affinity.

The majority of complications associated with bleomycin/pingyangmycin were mild, predominantly consisting of minor local adverse events and systemic inflammatory reactions with the exception of two cases of airway edema. Pingyangmycin exhibits low systemic toxicity as a chemotherapeutic agent. Pulmonary fibrosis, the most feared complication, has only been reported in some oncology patients receiving high cumulative intravenous doses. Both experimental and clinical studies have shown that its pulmonary toxicity is dose‐dependent. Reports indicate a notable risk of pulmonary fibrosis with systemic bleomycin doses above 160 mg, rising to 3%–5% when the total exceeds 450 mg, while lower doses carry minimal risk. A systematic review and meta‐analysis including 1406 patients who received intralesional bleomycin injections reported no instances of acute toxicity or pulmonary fibrosis in children, with cumulative doses ranging from 0.3 to 39 mg [101]. However, despite adhering to these dosage ranges, Alexander L. Cho et al. reported a case of fatal pulmonary toxicity in a 15‐month‐old girl following intralesional bleomycin injection for a macrocystic lymphatic malformation of the left cheek [102].

Méndez‐Echevarría A. et al. also described a case of severe acute pulmonary toxicity in a 5‐year‐old girl following a second low‐dose intralesional bleomycin injection for a cervical low‐flow venous malformation, despite the absence of known risk factors. The patient received prompt treatment with intravenous methylprednisolone, subsequently improved, and was discharged without any sequelae [103].

Similarly, Gasciunas A. et al. reported a case of acute lung injury in a 4‐year‐old boy following intralesional bleomycin sclerotherapy for a venous malformation in the left lower limb. The patient was managed with intravenous and oral corticosteroids, and at 1‐year follow‐up, he remained clinically stable with adequate respiratory function, although imaging continued to show residual pulmonary involvement [57]. No cases of pulmonary toxicity emerged in our review. However, given the reported case reports, caution is warranted when interpreting these findings. Clinicians treating these patients should remain vigilant for this complication in any patient presenting with respiratory symptoms after bleomycin sclerotherapy. Early recognition of pulmonary toxicity allows prompt management and may prevent permanent lung damage.

For vascular malformation treatment, total Pingyangmycin doses are usually under 100 mg, and no systemic toxicities affecting the liver and kidneys have been documented to date [14]. Similarly, in this review these complications were not observed.

Polidocanol demonstrates a highly favorable safety profile, with no severe adverse events reported. Specifically, no cardiovascular, visual, or hearing complications emerged in this systematic review. However, Marrocco‐Trischitta et al. described episodes of cardiac arrest in pediatric patients treated with this agent, underscoring the importance of cautious use in children [104]. Cui L. et al. reported a case of vision loss and hearing impairment in a 3‐year‐old boy following polidocanol foam sclerotherapy for a venous malformation on the cheek near the left orbit. The authors suggested that the complications may have resulted from extensive facial venous thrombosis extending into the orbital and petrosal venous networks due to excessive sclerosant injection. Potential mechanisms causing vision loss included inadvertent flow of sclerosant into the central retinal or posterior ciliary arteries causing obstruction and hemorrhage; orbital tissue swelling from tenonitis or a chronic allergic reaction to the sclerosant compressing ocular vessels; and a possible systemic toxic effect, given the concurrent hearing impairment, although vascular inflammation was not observed on imaging [105]. Similarly, Matsuo reported an 18‐year‐old patient afflicted with blindness caused by central retinal artery and posterior ciliary artery occlusion as a consequence of sclerotherapy with a polidocanol injection for a glabellar hemangioma [106].

Our results indicate that the two most commonly used agents for the treatment of lymphatic malformations are doxycycline and OK‐432.

Doxycycline has been widely applied in the management of malignant effusion pleurodesis and in the treatment of postoperative lymphoceles. In both settings, it has consistently demonstrated safety and efficacy, with only minimal adverse effects reported [107, 108].

Doxycycline is inexpensive, widely accessible, and generally well tolerated. Most published studies evaluating doxycycline for LM sclerotherapy have reported good to excellent short‐term outcomes with low toxicity rates [109, 110, 111]. However, evidence on medium‐ and long‐term clinical efficacy remains limited.

A potential long‐term concern associated with the use of tetracycline‐derived antibiotics is their possible impact on dental development in children. However, it should be emphasized that such adverse effects have primarily been reported in cases of prolonged administration and are generally restricted to fetal exposure in the first 6 months of life. No instances of dental developmental anomalies were reported among the patients included in this study. In our review, we identified a single case of transient nerve deficit following bleomycin sclerotherapy. Additionally, Johnston D. R. et al. reported a case of new‐onset dysphagia and pulmonary aspiration in an infant after sclerotherapy for a complex cervical venolymphatic malformation, resulting from injury to the vagus or laryngeal nerve [112]. Wang K. L. et al. also reported a similar case resulting in silent aspiration, which subsequently resolved [113]. These observations highlight the importance of carefully considering the anatomical location of the lesion when selecting the appropriate sclerosing agent, allowing the potential impact of even transient nerve deficits to be carefully weighed. Also, caution is advised when using doxycycline with diabetic patients as it may cause severe transient hypoglycemia.

Published studies have demonstrated the effectiveness of OK‐432 in the treatment of lymphatic macrocystic lesions, with regression achieved in up to 96% of cases [114, 115]. The efficacy of OK‐432 sclerotherapy in lymphatic malformations varies across centres and practitioners. It is well established that macrocystic lesions respond more favorably than microcystic ones [116, 117], suggesting that multiple factors influence the therapeutic success of OK‐432 in this setting. OK‐432 induces a localized inflammatory reaction sufficient to damage the endothelium and promote regression of the lymphatic malformation. Following sclerotherapy, patients frequently report symptoms related to this inflammatory process, including local pain, tenderness, swelling, erythema at the injection site, and low‐grade fever. The occurrence of such post‐treatment inflammatory signs has been identified as a relevant predictor of therapeutic success [27].

Our findings suggest that the safety profile is comparable between doxycycline and picibanil—defined as the absence of major complications. However, doxycycline tolerability is much higher, as it causes minor to no swelling and/or pain, all aspects that can be troublesome with OK‐432.

In the studies analyzed in this review, treatment efficacy outcomes were not consistently reported. When they were provided, the criteria varied, including size reduction (assessed through photographic evaluation and/or imaging) and improvement in quality of life measured using questionnaires. Since specific criteria for assessing the efficacy of these agents, nor defined response cut‐off values, have not yet been established, this review did not include an analysis of the therapeutic effectiveness of the various sclerosing agents. A large‐scale prospective comparative trial is essential to definitively establish the true therapeutic effectiveness of these sclerosing agents.

This systematic review is inherently limited by the overall low quality of the included studies. There was substantial variability in study designs and treatment protocols, and control or comparator groups were largely absent. Therefore, the findings should be interpreted cautiously and primarily regarded as a basis for generating hypotheses for future research. Despite these limitations, the review offers an up‐to‐date synthesis of the literature, summarizing the available evidence to guide clinical decision‐making. Subgroup analyses for lymphatic and venous malformations could not be performed due to the small number of patients and lack of subgroup‐specific outcome data. Similarly, comparisons of sclerosing agents across relevant subgroups, including malformation subtypes or anatomical locations, were not feasible. Furthermore, focusing solely on the safety profile, in the absence of robust efficacy data, represents a significant constraint when selecting the most appropriate therapeutic strategy.

## 5 Conclusions

This review provides an overview of the adverse effects associated with all sclerosant agents reported in the literature. Further research is necessary to correlate clinical outcomes with adverse effects to identify the agent with the most favorable profile. To achieve more robust assessments, prospective randomized studies are required, although such studies may be challenging to conduct.

## Funding

The authors have nothing to report.

## Data Availability

The data that support the findings of this study are available from the corresponding author upon reasonable request.
